# Impaired renal function in a rural Ugandan population cohort

**DOI:** 10.12688/wellcomeopenres.14863.3

**Published:** 2019-05-20

**Authors:** Robert Kalyesubula, Jeffrey P. Hau, Gershim Asiki, Billy Ssebunya, Sylvia Kusemererwa, Janet Seeley, Liam Smeeth, Laurie Tomlinson, Robert Newton

**Affiliations:** 1 MRC/UVRI and LSHTM Uganda Research Unit, Entebbe, Uganda; 2 Department of Physiology, Makerere University College of Health Sciences, Kampala, Uganda; 3 Non-Communicable Disease Epidemiology, Department of Epidemiology and Population Health, London School of Hygiene and Tropical Medicine, London, UK; 4 African Population and Health Research Center, Nairobi, Kenya; 5 Global Health and Development Department, London School of Hygiene and Tropical Medicine, London, UK; 6 University of York, York, UK

**Keywords:** Kidney disease, population cohort, epidemiology, prevalence

## Abstract

**Background: **Kidney disease is an important cause of morbidity and mortality globally. However, there are limited data on the prevalence of impaired kidney function in sub-Saharan Africa. We aimed to determine the prevalence of reduced kidney function and associated factors in a rural Ugandan population.

**Methods: **We undertook a study of a representative sample of the General Population Cohort in South-western Uganda. We systematically collected data on cardiovascular disease risk factors, anthropometric measurements and blood tests including haemoglobin, HIV, HbA1c and serum creatinine.  The estimated glomerular filtration rate (eGFR) was calculated using the CKD-Epi equation, without the race component of the equation.

**Results: **A total of 5,979/6,397 (93.5%) participants had valid creatinine results. The mean age was 39 years (Range:16-103 years) and 3,627 (60.7%) were female. HIV prevalence was 9.7% and about 40% of the population were pre-hypertensive or hypertensive. The mean serum creatinine level was 0.75 mg/dl (95% CI 0.74–0.75), and the average eGFR was 109.3 ml/min/1.73 m ^2^ (95% CI 108.8–109.9). The overall prevalence of eGFR <60 ml/min/1.73 m ^2^ was 1.64% (98/5,979) (95% CI 1.34–1.99).  Additionally, 4,792(80.2%) were classified as normal eGFR (≥90 ml/min/1.73 m ^2^), 1,089(18.2%) as low eGFR (60–89 ml/min/1.73 m ^2^), 91(1.52%) as moderately reduced eGFR (30–59 ml/min/1.73 m ^2^), 4(0.07%) as severely reduced eGFR (15-29 ml/min/1.73 m ^2^), and 3(0.05%) classified as having kidney failure (eGFR<15 ml/min/1.73 m ^2^).  When age-standardised to the WHO Standard Population the prevalence of eGFR<60 ml/min/1.73 m ^2^ was 1.79%. Age above 35 years and the presence of hypertension (OR 2.86, 95% CI 1.15-7.08) and anaemia (OR 2.14, 95% CI 1.12-4.09) were associated with eGFR<60 ml/min/1.73 m ^2^.

**Conclusion: **In a systematic survey of people in rural Uganda, we found a substantial proportion had eGFR<60 ml/min/1.73 m ^2^. More population based studies are needed to further characterize kidney disease in sub-Saharan Africa.

## Introduction

Chronic kidney disease (CKD) is an under-recognized non-communicable disease, associated with a high morbidity and mortality. It is estimated that one in ten people are living with kidney disease worldwide and the prevalence may be higher in low-income countries such as Uganda ^1, 2^. However, as shown in a recent systematic review the quality of data is often poor, frequently using convenience samples in high-risk populations ^3^. Furthermore, only 2% of the studies included in this review used the CKD-EPI equation for calculation of estimated glomerular filtration rate (eGFR) which, based on limited data, has been found to be the best estimate of population CKD prevalence ^3, 4^. There is little distinction between impaired renal function and chronic kidney diseases in most of the studies from sub-Saharan Africa where these two are used interchangeably ^3^. It is thus more appropriate to use impaired renal function rather than CKD when creatinine and albuminuria are measured once with no repeated measure to comfirm chronicity. Community-based studies of the prevalence of impaired renal function have shown marked variation in results. Among people living with HIV/AIDS, estimates range from 0.7% in Rakai, Central Uganda, 14.4% in Gulu, Northern Uganda, 26.5% in Zambia to 41.3% in Tanzania ^5– 8^. Among HIV-negative populations, estimates range from 2.5% in Wakiso, Central Uganda to 26.5% in Tanzania ^8, 9^. Hospital-based studies from a National Referral Hospital in Uganda show that most patients with kidney disease are young and have advanced disease by the time of presentation ^10^. Thus, in sub-Saharan Africa estimates of kidney disease prevalence vary widely depending on the methods used to determine renal function and the population studied, in particular the age distribution ^8, 11– 19^.

Globally, among the known key risk factors for CKD are diabetes mellitus, hypertension and infections such as HIV. Hypertension and HIV are important problems in Uganda with hypertension prevalence estimated to be 26.4% ^20^ and rising among those with HIV-infection ^21^. However, the prevalence of diabetes mellitus is low compared to high-income countries at 2% ^22^. Moreover, some studies have also highlighted differences in the prevalence of impaired renal function between urban and rural areas in Africa. A study from Cameroon found the overall prevalence of CKD to be 13.2%: 14.1% and 10.9% among rural and urban dwellers, respectively ^23^. Late diagnosis, along with limited health care leading to poor control of hypertension and diabetes may be possible drivers of a higher prevalence in rural populations.

Therefore, we aimed to determine the prevalence and associations of impaired renal function among a representative sample of a rural area of Uganda, within an existing population cohort using high quality sampling methods.

## Methods

### Study design and setting

The General Population Cohort (GPC) was established in 1989, by the United Kingdom Medical Research Council and the Uganda Virus Research Institute, in Kalungu District, South-western Uganda ^24^. The cohort was initially established to examine prevalence, incidence, risk factors and trends of infection with HIV in a rural African population. More recently, research activity has broadened to include the epidemiology and genetics of other communicable and of non-communicable diseases, including cancer, cardiovascular disease and diabetes ^24, 25^. In brief, the GPC is a community-based open cohort study of residents of 25 neighbouring villages within one-half of a sub-county, lying about 40 km from Lake Victoria. The population is scattered across the countryside in villages defined by administrative boundaries, with a few concentrated in small trading centres. The population under survey includes approximately 22,000 people, less than half of whom are more than 13 years of age. The cohort is dynamic with new births, deaths and migration reported at each round of follow-up. Data are collected through an annual census, an annual questionnaire and serological survey from 1989–2011 and a biennial questionnaire and serological survey thereafter. Details of sexual behaviour, medical, sociodemographic and geographic factors are recorded. Blood specimens are obtained at each biennial survey. Serum is tested for HIV-1 and the remainder is stored at -80°C. Since 1989, the seroprevalence of HIV has remained relatively stable in this population, with about 8% of participants infected; in recent years, prevalence has risen slightly, with the roll out of antiretroviral therapy and consequent improvements in survival.

All eligible participants were evaluated for the study with an acceptance rate of 98%. A total of 6,397 participants were indentified in the two rounds of the GPC (2011–2012 and 2014–2015) with 5,979 (93.5%) individuals having valid creatinine results. The 418 who did not have valid creatinine did not differ significantly from those selected. Variables used for analysis were extracted from two rounds, and participants’ information gathered from questionnaire and laboratory data of the survey rounds were linked by unique identifiers. For adults (18+ years for males and 16+ years for females), variables used to develop a socioeconomic score (SES), smoking status, alcohol consumption, fruit and vegetable intake and results of Hepatitis B and C tests were derived from the 2011–2012 survey round. Variables associated with participant’s eGFR, age, maximum education level, current marital status, history of stroke, body mass index (BMI) and HIV status were based on the 2014–2015 survey round.

### Data collection

Data collected from the GPC questionnaire regarding sexual behaviour and lifestyle factors were self-reported ( Supplementary File 1). Anthropometric measurements and blood tests were performed by trained interviewers/nurses using calibrated instruments and following standard operating procedures. We adapted the World Health Organization (WHO) STEP-wise approach to surveillance questionnaire to obtain socio-demographic characteristics, lifestyle (diet, tobacco, and alcohol consumption), medical history and biophysical measurements. Blood pressure was measured using a digital sphygmomanometer (Omron M4-1). The participant had to be in a sitting position and the mean of the second and third readings taken at 5-minute intervals was used for analysis. Body weight was measured using the Seca 761 mechanical scales and body height was measured using a stadiometer to the nearest 1 kg and 0.1 cm, respectively. Both scales were calibrated according to manufacturer guidelines weekly.

### Laboratory tests

Blood tests for haemoglobin, HIV screening, HbA1c, hepatitis B and C viruses, as well as the creatinine level were performed. Venous blood was tested for haemoglobin level using CT -5 Coulter Ac.T 5diff AL (Autoloader) [Beckman Coulter, North America]. HIV testing was performed using an approved national algorithm ^26^. Hepatitis B surface antigen, Hepatitis C antibody and creatinine level were tested using a Cobas e 601 Auto Analyzer (Roche Diagnostics, North America). Creatinine was measured using the Jaffe method traceable to an isotope dilution mass spectrometry method ^27^. The MRC/UVRI Entebbe laboratories currently have laboratory accreditation through ISO 15189 of the Kenya Accreditation Service, and are enrolled in external quality control programs for South Africa, America, Australia and the United Kingdom.

### Definitions and classification

Each participant’s SES was derived from conducting principal component analysis (PCA) using variables relating to household infrastructure and property ownership. Urbanicity score used in this study was derived from a previous study using information from the Round 22 survey ^28^. BMI was classified according to WHO categories (weight/height ^2^: kg/m ^2^): underweight (<18.5 kg/m ^2^), normal weight (18.5–24.9 kg/m ^2^), overweight (25.0–29.9 kg/m ^2^) and obese (>30.0 kg/m ^2^). Blood pressure (BP) classification was derived from the National Institute of Health guidelines: Pre-Hypertension was defined as having a systolic BP greater than 120mmHg but less than 140 mmHg, and a diastolic BP greater than 80 mmHg but less than 90 mmHg. Hypertension was defined as having a diastolic BP greater than or equal to 90 mmHg, systolic BP greater than or equal to 140 mmHg or being on treatment for high BP. Anaemia was defined as having haemoglobin levels less than 130 g/l in men, 120 g/l in non-pregnant women, and 110 g/l in pregnant women. Diabetes mellitus was diagnosed by either having HbA1c >6.5%, through self-reported measures of being previously diagnosed with diabetes, or by current treatment for diabetes.

### Classification of renal function

The estimated glomerular filtration rate (eGFR) was calculated using the CKD-Epi equation, without use of the coefficient for African Americans ^29^. Impaired renal function was divided into five categories analogous to CKD stages, based on the National Kidney Foundation guidelines (without including proteinuria) as: normal eGFR (≥90 ml/min/1.73 m ^2^); low eGFR (60–89 ml/min/1.73 m ^2^); moderately reduced eGFR (30–59 ml/min/1.73 m ^2^); severely reduced eGFR (15–29 ml/min/1.73 m ^2^); and kidney failure (eGFR <15 ml/min/1.73 m ^2^) ^30^. We have used impaired renal function for the study because we did not have a second creatinine after 3 or more months or measures of urinary protein excretion to comfirm CKD.

### Statistical analysis

Baseline characteristics were tabulated stratified by sex. The prevalence of impaired renal function was also age standardised using the WHO world population as the reference.

We used logistic regression to estimate odd ratios (OR), along with its 95% confidence intervals (95% CIs), to identify potential factors independently associated with CKD. A forward step-wise approach was used in developing our multivariable model adjusting for age, sex, and all independent predictors of CKD.

We also conducted a secondary analysis to compare participants with eGFR<60 ml/min/1.73 m ^2^ to those with normal renal function excluding individuals in the low eGFR category. The population attributable fraction (PAF) of impaired renal function was estimated for hypertension, and anaemia using the adjusted odds ratios from the final multivariable model.

All statistical analyses were performed using STATA 13 SE (Stata Corp, Texas, USA).

### Ethical considerations

All study participants gave written informed consent to participate in the study. The study was approved by Uganda Virus Research Institute Research and Ethics Committee (UVRI-REC) and the Uganda National Council for Science and Technology (UNCST).

## Results

### Baseline characteristics of study participants

A total of 6,397 individuals participated in the Round 24 GPC survey in 2014–2015 and 5,979 (93.5%) individuals had valid creatinine test results. The average age of study participants was 39 years (range: 16 years to 103 years), consisting of 3,626 (60.7%) females. The majority of patients had primary-level education (60.4%). HIV prevalence was 9.7% (males: 8.4%, females: 10.5%) within this study population, and about 40% of the population was classified as pre-hypertensive. The mean serum creatinine level of the study population was 66.3 mmol/l (95% CI 65.4–66.3), and using the CKD-EPI equation, the average eGFR was 109.3 ml/min/1.73 m ^2^ (95% CI 108.8–109.9) ( Table 1).

**Table 1.** Characteristics of participants with creatinine results from Survey round 24 among a general population cohort in rural Uganda (N=5,979).

Variable	Male, n (%)	Female, n (%)	Total, n (%)
Age Group			
<35	1,033 (43.90)	1,703 (46.97)	2,736 (45.77)
35–44	460 (19.55)	721 (19.88)	1,181 (19.74)
45–54	378 (16.02)	507 (13.98)	884 (14.79)
55–64	244 (10.37)	336 (9.27)	580 (9.70)
65–74	138 (5.86)	231 (6.37)	369 (6.17)
75+	101 (4.29)	128 (3.53)	229 (3.83)
Max Education			
None	137 (5.82)	394 (10.87)	531 (8.88)
Primary	1,488 (63.24)	2,122 (58.52)	3,610 (60.38)
Secondary	570 (24.21)	946 (26.09)	1,516 (25.35)
Higher Level	158 (6.71)	164 (4.52)	322 (5.38)
Currently Married **			
No	357 (20.62)	1,075 (36.68)	1,432 (30.72)
Yes	1,373 (79.36)	1,856 (63.32)	3,229 (69.28)
Urbanicity * ^1^			
Quartile 1	513 (28.71)	756 (26.31)	1,259 (27.24)
Quartile 2	468 (26.19)	733 (25.86)	1,201 (25.98)
Quartile 3	436 (24.40)	697 (24.59)	1,133 (24.51)
Quartile 4	370 (20.71)	659 (23.25)	1,029 (22.26)
SES * ^2^			
Lower	565 (35.76)	819 (32.79)	1,384 (33.94)
Middle	521 (33.04)	833 (33.35)	1,354 (33.23)
Upper	493 (31.20)	846 (33.87)	1,339 (32.83)
BMI ^3^ **			
Normal weight	1,786 (76.47)	2,290 (65.84)	4,076 (70.11)
Underweight	407 (17.42)	302 (8.68)	709 (12.19)
Overweight	122 (5.22)	648 (18.63)	770 (13.24)
Obese	21 (0.90)	238 (6.84)	259 (4.45)
Blood Pressure * ^4^			
Normal	668 (40.44)	1,235 (48.82)	1,903 (45.51)
Pre-Hypertension	719 (43.52)	944 (37.28)	1,663 (39.75)
Hypertension	265 (16.04)	352 (13.90)	617 (14.75)
HIV Status **			
Negative	2,150 (91.57)	3,242 (89.51)	5,392 (90.32)
Positive	198 (8.43)	380 (10.49)	578 (9.68)
Hepatitis B *			
Negative	1,588 (96.48)	2,479 (98.10)	4,067 (97.46)
Positive	58 (3.52)	48 (1.90)	106 (2.54)
Hepatitis C *			
Negative	1,582 (96.17)	2,439 (96.52)	4,021 (96.38)
Positive	63 (3.83)	88 (3.48)	151 (3.62)
Anaemia * ^5^			
Negative	1,078 (86.87)	1,583 (83.40)	2,661 (84.77)
Positive	163 (13.13)	315 (16.60)	478 (15.23)
Diabetes * ^6^			
No	1,603 (97.74)	2,467 (97.94)	4,070 (97.53)
Yes	37 (2.26)	52 (2.06)	89 (2.14)
Current smoking status *			
Not current smoker	1,301 (78.80)	2,478 (97.87)	3,779 (90.34)
Non-daily smoker	83 (5.03)	17 (0.67)	100 (2.39)
Daily smoker	267 (16.17)	37 (1.46)	304 (7.27)
Alcohol consumption *			
Never drinkers	831 (54.64)	1,589 (69.27)	2,420 (63.43)
No alcohol in past 30 days	90 (5.92)	250 (10.90)	340 (8.91)
Alcohol in past 30 days	600 (39.45)	455 (19.83)	1,055 (27.65)

^*^Variables from a previous round (R22) of the GPC where total number of participants may vary: Urbanicity (n=4,622), SES (n=4,077), Blood Pressure (BP) (n=4,184), Hepatitis B (n=4,173), Hepatitis C (n=4,172), smoking status (n=4,183), alcohol consumption in the last 30 days (n=3,815), and anaemia (n=3,139). ^1^Urbanicity score derived from Riha *et al.* (2014). ^2^Socio-economic Score (SES) derived from conducting Principle Component Analysis (PCA) on a statistical software using variables relating to household infrastructure and property ownership ^3^Body Mass Index (BMI) Classification according to WHO (weight/height ^2^: kg/m ^2^): Underweight (<18.5 kg/m ^2^), Normal weight (18.5–24.99 kg/m ^2^), Overweight (25.0–29.99 kg/m ^2^), Obese (>30.0 kg/m ^2^). ^4^BP classification derived from the National Institute of Health guidelines: Pre-Hypertension was defined as having a systolic BP >120 mmHg but <140 mmHg, and a diastolic BP >80 mmHg but <90 mmHg. Hypertension was defined as having a systolic BP ≥90 mmHg, diastolic BP ≥140 mmHg. ^5^Anaemia was defined as having haemogloblin levels less than 130 g/L in men, 120 g/L in non-pregnant women, and 110 g/L in pregnant women. Only 2,064 individuals had anaemia results from the R24 of the GPC ^6^Diabetes was defined as having HbA1C >6.5%, or being previously diagnosed with diabetes, or are currently on treatment for diabetes. **Variables in R24 with missing individuals: Currently Married (n=4,661), BMI (n=5,814), HIV (n=5,970)

### Prevalence of impaired renal function

**Figure 1.**
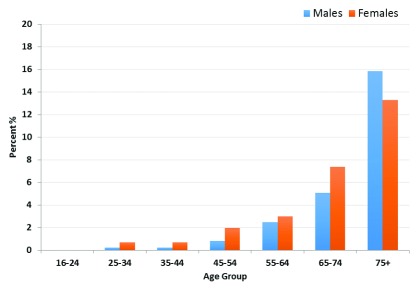
Prevalence of estimated glomerular filtration rate <60 ml/min/1.73 m ^2^ by age group among a rural Ugandan cohort.

The overall prevalence of eGFR <60 ml/min per 1.73 m ^2^ was 1.6% (95% CI 1.34–1.99). Of the respondents, 4,792 (80.2%) were classified as normal, 1,089 (18.2%) as low eGFR, 91 (1.5%) as moderately reduced eGFR, 4 (0.1%) as severely reduced eGFR, and 3 (0.1%) classified as having kidney failure ( Figure 1, Table 2). The prevalence of impaired renal function among those over the age of 16, age-standardised to the WHO population, was 1.8%.

**Table 2.** Mean serum creatinine and categories of estimated glomerular filtration rate (eGFR) in the general population cohort.

Variables	Mean (95% CI)
Measurements	
Serum creatinine (mg/dl)	0.75 (0.74–0.75)
eGFR (ml/min/1.73m ^2^)	
CKD-EPI equation * ^1^	109.3 (108.8–109.9)
MDRD equation *	106.2 (105.4–107.1)
	*Individuals n(%)*
**Category of level of eGFR**	
Normal eGFR (≥90 ml/min per 1.73 m ^2^)	4,792 (80.15)
Low eGFR (60–89 ml/min per 1.73 m ^2^)	1,089 (18.21)
Moderately reduced eGFR (30–59 ml/min per 1.73 m ^2^)	91 (1.52)
Severely reduced eGFR (15–29 ml/min per 1.73 m ^2^)	4 (0.07)
Kidney Failure (eGFR <15 ml/min per 1.73 m ^2^)	3 (0.05)

*The CKD-EPI eGFR calculations were used as the primary outcomes in this study; the MDRD equation was used to contrast the difference between the two equations. ^1^The coefficient for black race was omitted while using this equation.

### Factors associated with eGFR <60 ml/min per 1.73 m ^2^

Age and sex adjusted associations with the presence of eGFR <60 ml/min per 1.73 m ^2^ are shown in Supplementary Table 1. In multivariable analysis, older age, hypertension (OR 2.86; 95% CI 1.15-7.08) and anaemia (OR 2.14 ; 95% CI 1.12-4.09) were independently associated with eGFR <60 ml/min per 1.73 m ^2^ ( Table 3).

**Table 3.** Final multivariable model of factors independently associated with estimated glomerular filtration rate <60 ml/min per 1.73m ^2^.

Variable	Adjusted OR (95% CI) ^1^
*Sex*	P=0.56
Male	*Reference*
Female	1.19 (0.64–2.24)
*Age Group*	P<0.001
<35	*Reference*
35–44	0.53 (0.05–5.14)
45–54	3.49 (0.86–14.09)
55–64	5.73 (1.47–22.25)
65–74	12.24 (3.27–45.82)
75 +	29.68 (7.99–110.19)
*Blood Pressure* * ^2^	P=0.05
Normal	*Reference*
Pre-Hypertension	1.92 (0.81–4.57)
Hypertension	2.86 (1.15–7.08)
*Anaemia* ^3^	P=0.02
Negative	*Reference*
Positive	2.14 (1.12–4.09)

*Variables from a previous round (R22) of the GPC where total number of participants may vary: Blood Pressure (n=3,039). ^1^Multivariable model adjusted for age, sex and all independent predictors of eGFR <60mls/min per 1.73 m ^2^. OR, odds ratio; 95% CI, 95% confidence interval. ^2^Blood pressure classification derived from the National Institute of Health guidelines: Pre-Hypertension was defined as having a systolic blood pressure greater than 120 mmHg but less than 140 mmHg, and a diastolic blood pressure greater than 80 mmHg but less than 90 mmHg. Hypertension was defined as having a systolic blood pressure (BP) greater than or equal to 90mmHg, diastolic BP greater than or equal to 140mmHg. ^3^Anaemia was defined as having haemoglobin levels less than 130 g/l in men, 120 g/L in non-pregnant women, and 110 g/l in pregnant women. Only 2,064 individuals had anaemia results from the R24 of the GPC

Age and sex adjusted associations of variables with the presence of eGFR of <90 mls/min/1.73m ^2^ are shown in Supplementary Table 2. In multivariable analysis, female sex (OR 1.56, 95% CI 1.27-1.93); older age, higher urbanicity score, being overweight or obese; having hypertension (OR 1.60, 95 % CI 1.22-2.11) and HIV-positive status (OR 1.55, 95% CI 1.13-2.04) were associated with impaired kidney function ( Supplementary Table 3).

Comparison of participants with eGFR <60 ml/min/1.73m ^2^ to those with eGFR >90 ml/min/1.73m ^2^ revealed that older age, hypertension and anaemia were independently associated with impaired renal function ( Supplementary Table 4).

The adjusted population attributable fraction of decreased renal function attributable to hypertension and anaemia was 26.4% and 12.8%, respectively.

## Discussion

We found a prevalence of eGFR <60 mL/min per 1.73 m ^2^ of 1.64% in this predominantly young rural community of Uganda with more than one-fifth of the study participants having eGFR <90 mL/min per 1.73 m ^2^. Impaired renal function was strongly associated with age, high blood pressure and anaemia.

Comparing different prevalence estimates of impaired renal function from studies across sub-Saharan Africa is challenging for many reasons. In a meta-analysis of CKD in sub-Saharan Africa by Stanifer *et al.* ^3^ the overall prevalence was 13.9% but the majority of the studies were conducted among patients with known risk factors for renal disease such as diabetes mellitus, HIV infection and hypertension. Furthermore, only 2% of the included studies used the CKD-EPI equation for calculation of eGFR although, based on limited data, it has been found to be the best estimate of population CKD prevalence ^3, 4^. The age structure of population varies widely between countries in sub-Saharan Africa making standardisation to a reference population crucial for comparisons between regions or countries. In addition, the prevalence of risk factors such as HIV infection vary substantially across and within countries. We found a lower prevalence of of eGFR <60 mL/min per 1.73 m ^2^ in this rural setting than would be expected according to previous studies ^3, 23^. This unexpected finding could be explained by the characteristics of the population under study. Over 66% of our study participants were less than 45 years of age yet CKD prevalence increases with age. The traditional risk factors for CKD like diabetes mellitus, hypertension, obesity, alcohol intake and smoking were low in our population (see Table 1).

We found that older age, hypertension and anaemia were associated with impaired renal function. Age is known to be strongly associated with eGFR ^3, 23, 29^. Hypertension is both a cause and a consequence of kidney disease, and in this cross-sectional survey it was not possible to tell whether the participants had hypertension as a cause or consequence of the kidney disease. However there has been a rise in reported levels of hypertension in Uganda, from 13.7% ^31^ in 1969 to 26.4% in 2015 ^20^. Anaemia is also often a consequence of kidney disease, or may be due to shared risk-factors such as other chronic diseases. We found that anaemia was associated with kidney disease, even for patients with eGFR <90 ml/min/1.73m ^2^, a level of kidney function at which a direct causal effect would not be anticipated.

We only measured creatinine on one occasion while two results of eGFR <60 mL/min per 1.73 m ^2^ more than 3 months apart are required for the formal definition of CKD. This may have led to an overestimate of the prevalence of impaired renal function. However, most large scale epidemiological surveys have also used one measurement of creatinine ^32^. In addition, our study was prospectively sampled from well people and are thus likely to be affected by a transient fall in eGFR associated with acute illness. This is in contrast to many studies using routinely collected data to define renal function where misclassification is likely if blood tests are measured during when patients are unwell ^33^. Even if we had two measures of creatinine we would not have been able to confidently assert that patients with eGFR <60 mL/min per 1.73 m ^2^ had ‘chronic kidney disease’ as the estimating equations are not validated in sub-Saharan Africa and the long-term outcome implications, on which the CKD categorisation was defined, are not yet understood in this setting. From the multivariable analysis hypertension and anaemia are likely manifestations of chronic kidney disease, so it is not unreasonable to presume that impaired kidney function is an estimate of CKD. It is also very difficult to have a repeat creatinine and urinalysis in community studies. More studies are needed to establish the utility of the second creatinine/urinary protein in establishing chronicity of kidney disease. There is also need to put this in the context of poorly resourced countries where patients are likely to be lost to follow up. The initial contact may be the only opportunity to diagnose them and put them into formal care with the aim of reducing progression to serious complications ^10^.

Factors which have been traditionally associated with kidney disease in high-income countries such as smoking, alcohol intake and obesity were not associated with the presence of eGFR <60 mL/min per 1.73 m ^2^ in this population. This may be because of the low prevalence of these factors in the community, or may suggest that the risk factors for CKD are different in this region. Indeed, other researchers have found that the majority of kidney disease in sub-Saharan Africa is not explained by traditional risk factors ^34^.

### Study strengths and limitations

This was a large community-based study conducted within a well-characterized population cohort. We used the CKD-Epi equation to determine eGFR, which is thought to be the best estimate of true GFR in sub-Saharan Africa. We measured a wide range of social and anthropometric factors, chronic diseases and biochemical measurements in a structured and validated manner. In addition, our prevalence estimates have been standardized to the WHO population to enable comparability with other studies across the world.

However, there were limitations, including lack of screening for urine abnormalities (proteinuria and hematuria) which could have led us to underestimate the prevalence of kidney disease. Newer classifications of CKD require measurement of proteinuria to define kidney disease ^4^. We only measured creatinine on one occasion while two results of eGFR <60 mL/min per 1.73 m ^2^ more than 3 months apart are required for the formal definition of CKD. This may have led to an overestimate of the prevalence of impaired renal function.

### Implications of the study

Interventions for end-stage renal disease are currently limited for most countries in sub-Saharan Africa with very poor access to dialysis and kidney transplantation ^9, 35^. This study has established a significant prevalence of impaired renal function, highlighting the need to focus efforts on preventive strategies to delay onset and slow progression of renal disease. However, marked uncertainty remains about how best to estimate GFR in black Africans. This highlights the importance of our ongoing prospective study to determine the best way to measure renal function in sub-Saharan Africa: http://blogs.lshtm.ac.uk/ark/.

## Conclusions

We found that approximately one in five adults in rural Uganda had abnormal function despite a low prevalence of diabetes and obesity. More population based studies are needed to further characterize kidney disease in sub-Saharan Africa.

## Data availability

Owing to data protection concerns, there are restrictions on access to the underlying data. The GPC database contains 25 years of longitudinal data sets on demographics and disease surveillance. All data (census, survey and laboratory) generated through the cohort are stored and curated at the MRC/UVRI and the LSHTM Research Unit. Data access for specific research purposes is possible and has been granted previously. For any data access inquiries, you may contact the director, MRC/UVRI and the LSHTM Research Unit or by email to mrc@mrcuganda.org or the corresponding author.

## Supplementary material

Supplementary Table 1. Factors associated with estimated glomerular filtration rate (eGFR) <60 ml/min per 1.73 m ^2^ among a general population cohort from rural Uganda.

Supplementary Table 2. Factors associated with estimated glomerular filtration rate (eGFR) <90 ml/min per 1.73 m ^2^ among a general population cohort from rural Uganda.

Supplementary Table 3. Final multivariable model of factors independently associated with estimated glomerular filtration rate (eGFR) <90 ml/min per 1.73 m ^2^ among a general population cohort from rural Uganda.

Supplementary Table 4. Final multivariable model of factors independently associated with CKD comparing individuals with estimated glomerular filtration rate (eGFR) ≥90 ml/min per 1.73 m ^2^ to individuals with eGFR <60 ml/min per 1.73 m ^2^.

Supplementary File 1. GPC questionnaire regarding sexual behaviour and lifestyle factors.
