# Supplementary material for: Impaired renal function in a rural Ugandan population cohort
Source: Wellcome Open Res. 2019 May 20;3:149. Originally published 2018 Nov 19. [Version 3] doi: 10.12688/wellcomeopenres.14863.3 (PMC6560494; doi:10.12688/wellcomeopenres.14863.3)
Supplement: Supplementary file 1 [file wellcomeopenres-3-16684-s0001.tgz › 939a1a22-8e10-40ec-a15f-494af3fa358d_Supplementary_table_1_Revised.docx]

**Supplementary Table 1:** **Factors associated with eGFR <60 mL/min per 1.73 m^2^ among a general population cohort from rural Uganda (N=5,979)**

|  | **Total Individuals**  **n (%)** | **Individuals with eGFR <60 mL/min/1.73 m^2^**  **n (%)** | **Unadjusted OR**  **(95% CI)^1^** | **Age and sex adjusted**  **OR (95% CI)^1^** |
| --- | --- | --- | --- | --- |
| *Sex* |  |  | P=0.37 | P=0.16 |
| Male | 2,353 (39.35) | 34 (1.45) | *Reference* | *Reference* |
| Female | 3,626 (60.65) | 64 (1.76) | 1.20 (0.79-1.83) | 1.35 (0.87-2.08) |
| *Age Group* |  |  | P<0.001 | P<0.001 |
| <35 | 2,736 (45.77) | 6 (0.22) | *Reference* | *Reference* |
| 35-44 | 1,181 (19.74) | 6 (0.51) | 2.32 (0.74-7.22) | 2.33 (0.75-7.25) |
| 45-54 | 884 (14.79) | 13 (1.47) | 6.79 (2.57-17.92) | 6.89 (2.61-18.19) |
| 55-64 | 580 (9.70) | 16 (2.76) | 12.90 (5.02-33.13) | 13.07 (5.09-33.58) |
| 65-74 | 369 (6.17) | 24 (6.50) | 31.65 (12.84-77.97) | 31.66 (12.85-78.01) |
| 75+ | 229 (3.83) | 33 (14.41) | 76.60 (31.71-185.03) | 78.28 (32.38-189.26) |
| *Max Education^**^* |  |  | P<0.001 | P=0.007 |
| None | 531 (8.88) | 15 (2.82) | *Reference* | *Reference* |
| Primary | 3,610 (60.38) | 76 (2.10) | 0.73 (0.42-1.29) | 2.00 (1.10-3.62) |
| Secondary | 1,516 (25.35) | 3 (0.20) | 0.04 (0.01-0.19) | 0.56 (0.15-2.10) |
| Higher Level | 322 (5.38) | 4 (1.24) | 0.43 (0.14-1.31) | 2.66 (0.82-8.65) |
| *Currently Married^**^* |  |  | P<0.001 | P=0.49 |
| No | 1,432 (30.72) | 55 (3.84) | *Reference* | *Reference* |
| Yes | 3,229 (69.28) | 40 (1.24) | 0.32 (0.21-0.48) | 0.84 (0.51-1.37) |
| *Urbanicity*^2^* |  |  | P=0.35 | P=0.59 |
| Quartile 1 | 1,259 (27.24) | 26 (2.07) | *Reference* | *Reference* |
| Quartile 2 | 1,201 (25.98) | 17 (1.42) | 0.68 (0.36-1.26) | 0.69 (0.37-1.31) |
| Quartile 3 | 1,133 (24.51) | 25 (2.21) | 1.07 (0.61-1.86) | 1.04 (0.59-1.86) |
| Quartile 4 | 1,029 (22.26) | 15 (1.46) | 0.70 (0.26-1.33) | 0.96 (0.49-1.87) |
| *SES*^3^* |  |  | P=0.64 | P=0.29 |
| Lower | 1,384 (33.94) | 24 (1.73) | *Reference* | *Reference* |
| Middle | 1,354 (33.23) | 30 (2.21) | 1.28 (0.74-2.20) | 1.54 (0.88-2.71) |
| Upper | 1,339 (32.83) | 25 (1.87) | 1.07 (0.61-1.89) | 1.33 (0.74-2.39) |
| *BMI^4**^* |  |  | P=0.05 | P=0.33 |
| Normal weight | 4,076 (70.11) | 56 (1.37) | *Reference* | *Reference* |
| Underweight | 709 (12.19) | 19 (2.68) | 2.01 (1.18-3.41) | 0.93 (0.53-1.62) |
| Overweight | 770 (13.24) | 17 (2.21) | 1.65 (0.93-2.85) | 1.72 (0.95-3.10) |
| Obese | 259 (4.45) | 4 (1.54) | 1.14 (0.41-3.19) | 1.10 (0.38-3.18) |
| *Blood Pressure*^5^* |  |  | P<0.001 | P=0.005 |
| Normal | 1,903 (45.51) | 12 (0.63) | *Reference* | *Reference* |
| Pre-Hypertension | 1,663 (39.75) | 33 (1.98) | 3.19 (1.64-6.20) | 2.10 (1.06-4.16) |
| Hypertension | 617 (14.75) | 39 (6.32) | 10.63 (5.53-20.45) | 2.98 (1.47-6.02) |
| *HIV Status^**^* |  |  | P=0.83 | P=0.12 |
| Negative | 5,392 (90.32) | 88 (1.63) | *Reference* | *Reference* |
| Positive | 578 (9.68) | 10 (1.73) | 1.07 (0.55-2.07) | 1.78 (0.88-3.58) |
| *Hepatitis B** |  |  | P=0.92 | P=0.60 |
| Negative | 4,067 (97.46) | 82 (2.02) | *Reference* | *Reference* |
| Positive | 106 (2.54) | 2 (1.89) | 0.93 (0.22-3.85) | 1.49 (0.34-6.48) |
| *Hepatitis C** |  |  | P=0.51 | P=0.12 |
| Negative | 4,021 (96.38) | 82 (2.04) | *Reference* | *Reference* |
| Positive | 151 (3.62) | 2 (1.32) | 0.64 (0.15-2.64) | 0.37 (0.08-1.59) |
| *Anaemia^6^* |  |  | P<0.001 | P=0.003 |
| Negative | 2,661 (84.77) | 33 (1.24) | *Reference* | *Reference* |
| Positive | 478 (15.23) | 21 (4.39) | 3.65 (2.09-6.38) | 2.47 (1.37-4.42) |
| *Diabetes^7­^* |  |  | P=0.14 | P=0.42 |
| No | 4,070 (97.53) | 80 (1.97) | *Reference* | *Reference* |
| Yes | 89 (2.14) | 4 (4.49) | 2.34 (0.84-6.55) | 1.59 (0.54-4.65) |
| *Current Smoking Status** |  |  | P=0.16 | P=0.51 |
| Not current smoker | 3,779 (90.34) | 71 (1.88) | *Reference* | *Reference* |
| Non-daily smoker | 100 (2.39) | 2 (2.00) | 1.06 (0.25-4.40) | 0.62 (0.14-2.72) |
| Daily smoker | 304 (7.27) | 11 (3.62) | 1.96 (1.02-3.74) | 1.37 (0.66-2.81) |
| *Alcohol Consumption** |  |  | P=0.12 | P=0.74 |
| Never drinkers | 2,420 (63.43) | 34 (1.40) | *Reference* | *Reference* |
| No alcohol in past 30 days | 340 (8.91) | 9 (2.65) | 1.90 (0.90-4.01) | 1.11 (0.51-2.42) |
| Alcohol in past 30 days | 1,055 (27.65) | 23(2.18) | 1.56 (0.91-2.66) | 0.83 (0.46-1.50) |

^*^Variables from a previous round (R22) of the GPC where total number of participants may vary: Urbanicity (n=4,622), SES (n=4,077), Blood Pressure (BP) (n=4,184), Hepatitis B (n=4,173), Hepatitis C (n=4,172), smoking status (n=4,183), alcohol consumption in the last 30 days (n=3,815), and anaemia (n=3,139). ^1^Urbanicity score derived from Riha et al (2014). ^2^Socio-economic Score (SES) derived from conducting Principle Component Analysis (PCA) on a statistical software using variables relating to household infrastructure and property ownership

^3^Body Mass Index (BMI) Classification according to WHO (weight/height^2^: kg/m2): Underweight (<18.5 kg/m^2^), Normal weight (18.5 – 24.99 kg/m^2^), Overweight (25.0 – 29.99 kg/m^2^), Obese (>30.0 kg/m^2^). ^4^BP classification derived from the National Institute of Health guidelines: Pre-Hypertension was defined as having a systolic BP >120mmHg but <140 mmHg, and a diastolic BP >80 mmHg but <90 mmHg. Hypertension was defined as having a systolic BP ≥90mmHg, diastolic BP ≥140mmHg. ^5^Anaemia was defined as having haemogloblin levels less than 130 g/L in men, 120 g/L in non-pregnant women, and 110 g/L in pregnant women. Only 2,064 individuals had anaemia results from the R24 of the GPC

^6^Diabetes was defined as having HbA1C >6.5%, or being previously diagnosed with diabetes, or are currently on treatment for diabetes. ^**^Variables in R24 with missing individuals: Currently Married (n=4,661), BMI (n=5,814), HIV (n=5,970)
