# Supplementary material for: Impaired renal function in a rural Ugandan population cohort
Source: Wellcome Open Res. 2019 May 20;3:149. Originally published 2018 Nov 19. [Version 3] doi: 10.12688/wellcomeopenres.14863.3 (PMC6560494; doi:10.12688/wellcomeopenres.14863.3)
Supplement: Supplementary file 2 [file wellcomeopenres-3-16684-s0002.tgz › 51af12b0-ffe9-444e-b221-6f94884742ba_Supplementary_table_2_Revised.docx]

**Supplementary Table 2:** **Factors associated with eGFR <90 mL/min per 1.73 m^2^ among a general population cohort from rural Uganda**

|  | **Total Individuals**  **N(%)** | **Individuals with**  **eGFR <90 mL/min/1.73 m^2^**  **N(%)** | |  | **Unadjusted OR**  **(95% CI)^1^** | **Age and sex adjusted OR (95% CI)^1^** |
| --- | --- | --- | --- | --- | --- | --- |
| *Sex* |  |  | |  | P<0.001 | P<0.001 |
| Male | 2,352 (39.34) | 400 (17.01) | |  | *Reference* | *Reference* |
| Female | 3,627 (60.66) | 787 (21.70) | |  | 1.35 (1.18,1.54) | 1.71 (1.46,2.01) |
| *Age Group* |  |  | |  | P<0.001 | P<0.001 |
| <35 | 2,736 (45.77) | 94 (3.44) | |  | *Reference* | *Reference* |
| 35-44 | 1,181 (19.74) | 186 (15.75) | |  | 5.25 (4.05,6.80) | 5.32 (4.11,6.90) |
| 45-54 | 884 (14.79) | 231 (26.13) |  | | 9.94 (7.07,12.82) | 10.34 (8.01,13.36) |
| 55-64 | 580 (9.70) | 248 (42.76) |  | | 20.99 (16.13,27.32) | 22.03 (16.90,28.73) |
| 65-74 | 369 (6.17) | 255 (60.98) |  | | 43.91 (32.75,58.88) | 45.51 (33.86,61.16) |
| 75+ | 229 (3.83) | 203 (88.65) |  | | 219.44 (138.92,346.63) | 240.87 (151,381.85) |
| *Max Education^**^* |  |  |  | | P<0.001 | P=0.002 |
| None | 531 (8.88) | 205 (38.61) |  | | *Reference* | *Reference* |
| Primary | 3,610 (60.38) | 778 (21.56) |  | | 0.43 (0.36,0.52) | 1.21 (0.95,1.55) |
| Secondary | 1,516 (25.35) | 147 (9.69) |  | | 0.17 (0.13,0.21) | 1.29 (0.95,1.75) |
| Higher Level | 322 (5.38) | 57 (17.70) |  | | 0.34 (0.24,0.47) | 2.21 (1.47,3.33) |
| *Currently Married^**^* |  |  |  | | P<0.001 | P=0.61 |
| No | 1,432 (30.72) | 529 (36.94) |  | | *Reference* | *Reference* |
| Yes | 3,229 (69.28) | 612 (18.95) |  | | 0.39 (0.34,0.45) | 1.04 (0.87,1.24) |
| *Urbanicity*^2^* |  |  |  | | P<0.001 | P=0.018 |
| Quartile 1 | 1,259 (27.24) | 264 (20.97) |  | | *Reference* | *Reference* |
| Quartile 2 | 1,201 (25.98) | 241 (20.05) |  | | 0.94 (0.77,1.14) | 0.96 (0.76,1.22) |
| Quartile 3 | 1,133 (24.51) | 294 (25.95) |  | | 1.32 (1.09,1.59) | 1.34 (1.06,1.68) |
| Quartile 4 | 1,029 (22.26) | 182 (17.67) |  | | 0.80 (0.65,0.99) | 1.18 (0.91,1.52) |
| *SES*^3^* |  |  |  | | P=0.69 | P=0.20 |
| Lower | 1,384 (33.94) | 314 (22.69) |  | | *Reference* | *Reference* |
| Middle | 1,354 (33.23) | 289 (21.34) |  | | 0.92 (0.77,1.10) | 1.08 (0.87,1.35) |
| Upper | 1,339 (32.83) | 297 (22.20) |  | | 0.97 (0.81,1.16) | 1.22 (0.98,1.51) |
| *BMI^4**^* |  |  |  | | P<0.001 | P<0.001 |
| Normal weight | 4,076 (70.11) | 714 (17.52) |  | | *Reference* | *Reference* |
| Underweight | 709 (12.19) | 176 (24.82) |  | | 1.55 (1.28,1.87) | 0.68 (0.53,0.87) |
| Overweight | 770 (13.24) | 193 (25.06) |  | | 1.57 (1.31,1.88) | 1.45 (1.17,1.80) |
| Obese | 259 (4.45) | 86 (33.20) |  | | 2.34 (1.78,3.06) | 1.97 (1.44,2.70) |
| *Blood Pressure*^5^* |  |  |  | | P<0.001 | P<0.001 |
| Normal | 1,903 (45.51) | 290 (15.24) |  | | *Reference* | *Reference* |
| Pre-Hypertension | 1,663 (39.75) | 402 (24.17) |  | | 1.77 (1.49,2.09) | 1.31 (1.08,1.59) |
| Hypertension | 617 (14.75) | 281 (45.47) |  | | 4.63 (3.79,5.67) | 1.56 (1.22,2.00) |
| *HIV Status^**^* |  |  |  | | P=0.046 | P=0.001 |
| Negative | 5,392 (90.32) | 1,050 (19.47) |  | | *Reference* | *Reference* |
| Positive | 578 (9.68) | 133 (23.01) |  | | 1.23 (1.00,1.51) | 1.47 (1.17,1.86) |
| *Hepatitis B** |  |  |  | | P=0.11 | P=0.63 |
| Negative | 4,067 (97.46) | 949 (23.33) |  | | *Reference* | *Reference* |
| Positive | 106 (2.54) | 18 (16.98) |  | | 0.67 (0.40,1.12) | 0.87 (0.49,1.53) |
| *Hepatitis C** |  |  |  | | P=0.084 | P=0.70 |
| Negative | 4,021 (96.38) | 923 (22.95) |  | | *Reference* | *Reference* |
| Positive | 151 (3.62) | 44 (29.14) |  | | 1.38 (0.96,1.97) | 0.91 (0.58,1.43) |
| *Anaemia^6^* |  |  |  | | P<0.001 | P=0.83 |
| Negative | 2,661 (84.77) | 504 (18.94) |  | | *Reference* | *Reference* |
| Positive | 478 (15.23) | 123 (25.73) |  | | 1.48 (1.18,1.86) | 0.97 (0.73,1.27) |
| *Diabetes^7­^* |  |  |  | | P=0.40 | P=0.17 |
| No | 4,070 (97.53) | 940 (21.10) |  | | *Reference* | *Reference* |
| Yes | 89 (2.14) | 24 (26.97) |  | | 1.22 (0.76,1.97) | 0.69 (0.39,1.19) |
| *Current Smoking Status** |  |  |  | | P=0.024 | P=0.23 |
| Not current smoker | 3,779 (90.34) | 859 (22.73) |  | | *Reference* | *Reference* |
| Non-daily smoker | 100 (2.39) | 33 (33.00) |  | | 1.67 (1.09,2.55) | 0.95 (0.57,1.58) |
| Daily smoker | 304 (7.27) | 81 (26.64) |  | | 1.23 (0.94,1.61) | 0.75 (0.54,1.04) |
| *Alcohol Consumption** |  |  |  | | P<0.001 | P=0.082 |
| Never drinkers | 2,420 (63.43) | 441 (18.22) |  | | *Reference* | *Reference* |
| No alcohol in past 30 days | 340 (8.91) | 107 (31.47) |  | | 2.06 (1.60,2.65) | 1.39 (1.02,1.88) |
| Alcohol in past 30 days | 1,055 (27.65) | 302 (28.65) |  | | 1.80 (1.52,2.13) | 0.97 (0.79,1.20) |

^*^Variables from a previous round (R22) of the GPC where total number of participants may vary: Urbanicity (n=4,622), SES (n=4,077), Blood Pressure (BP) (n=4,184), Hepatitis B (n=4,173), Hepatitis C (n=4,172), smoking status (n=4,183), alcohol consumption in the last 30 days (n=3,815), and anaemia (n=3,139). ^1^Urbanicity score derived from Riha et al (2014). ^2^Socio-economic Score (SES) derived from conducting Principle Component Analysis (PCA) on a statistical software using variables relating to household infrastructure and property ownership

^3^Body Mass Index (BMI) Classification according to WHO (weight/height^2^: kg/m2): Underweight (<18.5 kg/m^2^), Normal weight (18.5 – 24.99 kg/m^2^), Overweight (25.0 – 29.99 kg/m^2^), Obese (>30.0 kg/m^2^). ^4^BP classification derived from the National Institute of Health guidelines: Pre-Hypertension was defined as having a systolic BP >120mmHg but <140 mmHg, and a diastolic BP >80 mmHg but <90 mmHg. Hypertension was defined as having a systolic BP ≥90mmHg, diastolic BP ≥140mmHg. ^5^Anaemia was defined as having haemogloblin levels less than 130 g/L in men, 120 g/L in non-pregnant women, and 110 g/L in pregnant women. Only 2,064 individuals had anaemia results from the R24 of the GPC

^6^Diabetes was defined as having HbA1C >6.5%, or being previously diagnosed with diabetes, or are currently on treatment for diabetes. ^**^Variables in R24 with missing individuals: Currently Married (n=4,661), BMI (n=5,814), HIV (n=5,970)
