# Supplementary material for: Impaired renal function in a rural Ugandan population cohort
Source: Wellcome Open Res. 2019 May 20;3:149. Originally published 2018 Nov 19. [Version 3] doi: 10.12688/wellcomeopenres.14863.3 (PMC6560494; doi:10.12688/wellcomeopenres.14863.3)
Supplement: Supplementary file 3 [file wellcomeopenres-3-16684-s0003.tgz › 7c1cdea2-244a-40e6-9430-b0727d79392e_Supplementary_table_3_Revised.docx]

**Supplementary Table 3: Final multivariable model of factors independently associated with eGFR<90 mL/min per 1.73 m^2^ among a general population cohort from rural Uganda**

| **Variable** | **Adjusted OR (95% CI)^1^** |
| --- | --- |
| *Sex* | P<0.001 |
| Male | *Reference* |
| Female | 1.56 (1.27-1.93) |
| *Age Group* | P<0.001 |
| <35 | *Reference* |
| 35-44 | 4.35 (3.00-6.31) |
| 45-54 | 8.77 (6.14-12.54) |
| 55-64 | 16.95 (11.70-24.57) |
| 65-74 | 36.63 (24.45-54.89) |
| 75 + | 278.15 (148.28-521.79) |
| *Urbanicity*^2^* | P=0.013 |
| Quartile 1 | *Reference* |
| Quartile 2 | 0.96 (0.74-1.25) |
| Quartile 3 | 1.41 (1.09-1.82) |
| Quartile 4 | 1.18 (0.88-1.57) |
| *BMI^3**^* | P<0.001 |
| Normal weight | *Reference* |
| Underweight | 0.74 (0.55-0.99) |
| Overweight | 1.47 (1.13-1.91) |
| Obese | 1.78 (1.21-2.63) |
| *Blood Pressure** | P=0.002 |
| Normal | *Reference* |
| Pre-Hypertension | 1.19 (0.96-1.48) |
| Hypertension | 1.60 (1.22-2.11) |
| *HIV Status^**^* | P=0.006 |
| Negative | *Reference* |
| Positive | 1.55 (1.13-2.04) |

^**^Variables in R24 with missing individuals: Currently Married (n=4-661)- BMI (n=5-814)- HIV (n=5-970) * Variables from a previous round (R22) of the GPC where total number of participants may vary: Urbanicity (n=4-622)-SES (n=4-077)- Blood Pressure (n=4-184)

^1^OR denotes odds ratio; 95% CI denotes 95% confidence interval. ^2^Urbanicity score derived from Riha et al (2014). ^3^Body Mass Index (BMI) Classification according to WHO (weight/height^2^: kg/m^2^): Underweight (<18.5 kg/m^2^)- Normal weight (18.5 – 24.99 kg/m^2^)- Overweight (25.0 – 29.99 kg/m^2^)- Obese (>30.0 kg/m^2^). ^4^Blood pressure classification derived from the National Institute of Health guidelines: Pre-Hypertension was defined as having a systolic blood pressure greater than 120mmHg but less than 140 mmHg and a diastolic blood pressure greater than 80 mmHg but less than 90 mmHg. Hypertension was defined as having a systolic blood pressure (BP) greater than or equal to 90mmHg, diastolic BP greater than or equal to 140mmHg.
