# Supplementary material for: Impaired renal function in a rural Ugandan population cohort
Source: Wellcome Open Res. 2019 May 20;3:149. Originally published 2018 Nov 19. [Version 3] doi: 10.12688/wellcomeopenres.14863.3 (PMC6560494; doi:10.12688/wellcomeopenres.14863.3)
Supplement: Supplementary file 4 [file wellcomeopenres-3-16684-s0004.tgz › 5070c506-7287-4440-93ea-7296ed5cf5bf_Supplementary_table_4_Revised.docx]

**Supplementary Table 4: Final multivariable model of factors independently associated with eGFR <60 mL/min per 1.73 m^2^ where comparator group is individuals with eGFR ≥90 mL/min per 1.73 m^2^**

| **Variable** | **Adjusted OR (95% CI)^1^** |
| --- | --- |
| *Sex* | P=0.36 |
| Male | *Reference* |
| Female | 1.38 (0.67-2.81) |
| *Age Group* | P<0.001 |
| <35 | *Reference* |
| 35-44 | 0.59 (0.06-5.77) |
| 45-54 | 4.27 (1.05-17.27) |
| 55-64 | 8.84 (2.59-34.66) |
| 65-74 | 24.60 (6.43-94.08) |
| 75 + | 211.32 (51.54-867.69) |
| *Blood Pressure*^2^* | P=0.085 |
| Normal | *Reference* |
| Pre-Hypertension | 2.05 (0.82-5.10) |
| Hypertension | 2.90 (1.09-7.74) |
| *Anaemia^3^* | P=0.017 |
| Negative | *Reference* |
| Positive | 2.48 (1.19-5.15) |

^||^Individuals with a low kidney disease category (eGFR 60 - 89 mL/min per 1.73 m^2^) were excluded from this analysis. * Variables from a previous round (R22) of the GPC where total number of participants may vary: Blood Pressure (n=3-039). ^1^ Multivariable model adjusted for age, sex and all independent predictors of CKD. OR denotes odds ratio; 95% CI denotes 95% confidence interval. ^2^Blood pressure classification derived from the National Institute of Health guidelines: Pre-Hypertension was defined as having a systolic blood pressure greater than 120mmHg but less than 140 mmHg and a diastolic blood pressure greater than 80 mmHg but less than 90 mmHg. Hypertension was defined as having a systolic blood pressure (BP) greater than or equal to 90mmHg- diastolic BP greater than or equal to 140mmHg. ^3^Anaemia was defined as having haemogloblin levels less than 130 g/L in men, 120 g/L in non-pregnant women and 110 g/L in pregnant women. Only 2064 individuals had anaemia results from the R24 of the GPC.
