# Supplementary material for: Impaired renal function in a rural Ugandan population cohort
Source: Wellcome Open Res. 2019 May 20;3:149. Originally published 2018 Nov 19. [Version 3] doi: 10.12688/wellcomeopenres.14863.3 (PMC6560494; doi:10.12688/wellcomeopenres.14863.3)
Supplement: Supplementary file 5 [file wellcomeopenres-3-16684-s0000.tgz › e5ba6355-6b57-476a-85ce-d312e7926dbc_Supplementary_File_1.pdf]

MRC/UVRI SURVEY: Identification and characterization of chronic kidney disease in Uganda

PERSONAL IDENTIFIER INFORMATION

1. Consent obtained? |__| CONS

1 = yes, 2 = no
If no, do not continue

DDEXAM MDEXAM YDEXAM
2. Interviewer name & code no.………..…|__|__| INTCODE 3. Date of interview: |__|_| |__|__| |__|__|__|__|
dd mm yyyy

STICKER with participant's personal identifier information
Residence code: |__|__| VNO |__|__| BNO |__|__|__| HNO |__|__| STM

4. PARTICIPANT NAME: ....NAMEC |__|__|__|__|__|__|__|__|__| IDNO

5. SEX |__| 6. DOB |__|__| |__|__| |__|__|__|__|→ If year of birth unknown, ask or estimate age (years) |__|__| AGE
1 = M, 2 = F dd mm yy

6. What is your ethnicity?
   a. Muganda |__| ETN1
   b. Rwandese/ Barundi |__| ETN2
   c. Other |__| ETN3

7. What is your tribe? (use code list) |__| TRB

Information for survey clerks and data manager:
If person listed on Enumeration List, indicate any differences in age, name etc.
8. a. Revised name:……………………………………………………. RVNAME
   b. Revised date of birth: |__|__| DDOB |__|__| MDOB |__|__|__|__| YDOB or |__|__| AGER

Remarks: ....……………………………………………………………...

EDUCATION, OCCUPATION AND LIVELIHOOD

9. Are you in full-time education? |__| PSCH
1 = yes, 2 = no, 3 = don't know

If yes,
10. What level of education? |__| FTED
1 = pre-primary school; 2 = primary school; 3 = secondary school; 4 = higher education (e.g. college, university)
5 = vocational college

If no,
11. What is your source of livelihood? (use code list L) |__|__| OCCUP1
|__|__| OCCUP2
|__|__| OCCUP3
|__|__| OCCUP4

|__|__| OCCUP5

12. What level of education are you at (if still in education) or did you reach (if finished education)? |__|__| LEV
99 = nil; 18 = preprimary; 1-7 = P1-P7; 8-10 = J1-J3; 11-16 = S1- S6; 17 = college/university; 19 = vocational college

MARITAL STATUS
I'm going to ask you about your marital status. This means if you have ever regarded someone as your spouse.

13. Have you ever been married, that is, have you ever had someone you called your wife/husband? |__|EVM

1 =yes, 2 =no, 3 =don't know
If no, go to question 17

If yes,

14. How old were you when you first got married? State age (years) |__|__| AGEMG

PREGNANCY - for all female participants aged 13-49 (for male and 50+ female participants, go to question 20) FEMALE PARTICIPANTS

15. In the past 12 months, have you become pregnant? |__| PREGYR
1 = yes, 2 = no, 3 = don't know, 4 = not applicable
If no, go to question 20

If yes,
16. Did you attend antenatal clinic? 1 = yes, 2 = no, 3 = don't know, 4 = not applicable |__| ANCP

17. Have you ever had high blood pressure in pregnancy? |__| BPP
1 = yes, 2 = no, 3 = don't know

18. Have you ever had diabetes in pregnancy? |__| DMP
1 = yes, 2 = no, 3 = don't know

19. Have you ever had a miscarriage and or still birth? |__| MSB
1 = yes, 2 = no, 3 = don't know

HEALTH – for all participants

Interviewer: Please read this to the participant.
MRC has mainly been finding out about HIV. However it's also important to know about some other conditions in this community. So I'm now going to ask about some other conditions.

I am now going to ask you some questions about your health and lifestyle behaviours. This includes things like smoking, drinking alcohol, eating fruit and vegetables and physical activity. Let's start with tobacco.

TOBACCO USE

20. Do you currently smoke any tobacco products, such as cigarettes, cigars or pipes? |__|TOBAC1
1 = yes, 2 = no,

If no, go to question 22

21. Do you currently smoke tobacco products daily? |__|TOBAC2

1 = yes, 2 = no,
If yes go to question 23

22. In the past, did you ever smoke daily? |__|TOBAC3

1 = yes, 2 = no
If no, go to question 25

23. How old were you when you first started smoking daily? (Age in years) |__|__|TOBAC4
888 = don't know
If question 21 = 1 (you currently smoke), go to question 25)
If question 22 = 1 (you have smoked in the past, but do not currently smoke) go to question 24

24. How long ago did you stop smoking daily? |__|TOBAC5
1 = less than 4 weeks ago; 2 = more than 1 month but less than 12 months ago; 3 = more than one year, but less than 5 years ago; 4 = more than 5 years ago, 8 = don't know

25. Do you commonly chew any tobacco products?
1 = yes, 2 = no, 3 = don't know **If no go to Qn 27** |__|TOBAC6

26. If yes, how frequently?
1=Daily, 2 = 2-3x per week 3=once a week 4= once a month |__|TOBAC7

ALCOHOL CONSUMPTION

The next questions ask about the consumption of alcohol.

27. Have you ever consumed an alcoholic drink such as beer, wine, spirits, fermented cider or local products?
|__|ALC1

1 = yes, 2 = no,
If no, go to question 33

28. Have you consumed an alcoholic drink within the past 12 months?
|__|ALC2

1 = yes, 2 = no,
If no, go to question 32

29. During the past 12 months, how frequently have you had at least one alcoholic drink? |__|ALC3
1 = daily, 2 = 5-6 days per week, 3 = 1-4 days per week, 4 = 1-3 days per month, 5 = less than once a month

30. Have you consumed an alcoholic drink within the past 30 days? |__|ALC4

1 = yes, 2 = no
If no, go to question 32

31. During the past 30 days, on how many occasions did you have at least one alcoholic drink? Number |__|__|ALC5
88 = don't know

32. When was the last time you had an alcoholic drink? |__|ALC6
1 = today, 2 = yesterday, 3 = between 3 and 7 days ago, 4 = between 8 and 30 days ago

PHYSICAL ACTIVITY - WORK

33. Does your work involve activity that causes large increases in breathing or heart rate like *carrying or lifting heavy loads, very brisk walking, digging or construction work* for at least 10 minutes continuously? |__| PHYS1
1 = yes, 2 = no
If no, go to question 36

34. In a typical week, on how many days do you do these activities as part of your work?
Number of days |__|__| PHYS2

35. How much time do you spend doing these activities at work on a typical day?
Hours : minutes |__|__|**:**|__|__| PHYS3
Hrs mins

FRUITS, VEGETABLES, SALT AND WATER INTAKE

36. How many times each week do you eat fresh fruit or uncooked vegetable? Number |__|__| FV1
88= don't know

37. How often do you add salt to your food?
Rarely (<1x/wk) |__| SI1
Sometime (1-3x/wk) |__| SI2
Often (almost daily) |__| SI3
Frequently (multiple per day) |__| SI4

38. How much water do you drink in a day?
1☐ ≤ 1 L 2☐ 1-2 L 3☐ 2-3 L 4☐ 3-4 L 5☐ 4-5 L 6☐ ≥ 5 L |__|WI
88= don't know

FAMILY MEDICAL HISTORY

39. Does any family member (parents, siblings, or children) ever had or currently have any of the following diseases?
1 = yes, 2 = no 88= don't know
Diabetes |__| FDM
Hypertension |__| FHT
Heart disease |__| FHD

Dyslipidaemia |__| FDL
Chronic Kidney disease |__| FCKD

HISTORY OF RAISED BLOOD PRESSURE

40. Have you ever had your blood pressure measured by a doctor or other health worker? |__| HBP1
1 = yes, 2 = no

41. Have you ever been told by a doctor or other health worker that you have raised blood pressure or hypertension?
1 = yes, 2 = no |__| HBP2

If no, go to question 45

42. How long have you had raised blood pressure? Number |__|__| HBP3
88= don't know

43. Have you been told in the past 12 months that you have raised blood pressure or hypertension? |__| HBP4
1 = yes, 2 = no

44. During the past two weeks, have you been treated for raised blood pressure with drugs (medication) prescribed by a doctor or other health worker? |__| HBP5
1 = yes, 2 = no

HISTORY OF DIABETES

45. Have you ever had your blood sugar measured by a doctor or other health worker? |__| HD1
1 = yes, 2 = no

46. Have you ever been told by a doctor or other health worker that you have raised blood sugar or diabetes?
1 = yes, 2 = no |__| HD2
If no, go to question 50

47. How long have you had raised blood sugar? Number |__|__| HD3
88= don't know

48. Have you been told in the past 12 months that you have raised blood sugar or diabetes? |__| HD4
1 = yes, 2 = no **If no go to question 50**

49. Today, have you taken insulin or other drugs (medication) that have been prescribed by a doctor or other health worker for raised blood sugar? |__| HD5
1 = yes, 2 = no

HISTORY OF HIGH CHOLESTEROL

50. Have you ever had your cholesterol measured (by blood test) by a doctor or other health worker? |__| CHOLM
1 = yes, 2 = no

51. Have you ever been told by a doctor or other health worker that you have high cholesterol? |__| CHOLD
    1 = yes, 2 = no
    If no, go to question 54
52. Have you been told in the past 12 months that you have high cholesterol? |__| HCHOL

    1 = yes, 2 = no

53. During the past two weeks, have you been treated for high cholesterol with drugs (medication) prescribed by a doctor or other health worker? |__| CHOLTR
    1 = yes, 2 = no

HISTORY OF CHRONIC KIDNEY DISEASE

54. Have you ever suffered from Loin pain? |__|H LP
    1 = yes, 2 = no

55. Have you ever had repeated episodes of urinary tract infection? |__| HUTI
    1 = yes, 2 = no

56. Have you ever been told by a doctor or other health worker that you have Kidney disease?
    1 = yes, 2 = no |__| HCKD1
    If no, go to question 60

57. How long have you had kidney disease? Number |__|__| HCKD2
88= don't know

58. Have you been told in the past 12 months that you have Kidney Disease? |__| HCKD3
    1 = yes, 2 = no

59. Today, have you taken drugs (medication) that have been prescribed by a doctor or other health worker for Kidney disease? |__| CKDT
    1 = yes, 2 = no

HISTORY OF TAKING TRADITIONAL MEDICINE

60. Have you ever taken traditional medicine for any medical condition or for any other reasons including cultural rituals?
    1 = yes, 2 = no **If no go to question 63** |__| TRDE

61. Have you taken in the last 12 months traditional medicine for any medical condition or for any other reasons including cultural rituals?
1 = yes, 2 = no **If no go to question 63** |__| TRDY

62. When did you last take traditional medicine for any medical condition or for any other reasons including cultural rituals? |__| TRD
1= within last one month; 2= within last one week; 3= within less than one week
88= don't know

HISTORY OF TAKING TREATMENT FOR ANY CHRONIC DISEASE

63. Have you ever taken treatment for any chronic disease including HIV/AIDS? |__| TRT1
1 = yes, 2 = no **If no go to question 70**

If yes,

64. For what disease was it? (tick all that apply)
1= Diabetes, 2= High Blood pressure, 3= HIV/AIDS, 4= Chronic Kidney disease, 5=TB |__| TRT2
6= Heart disease, 7= Cancer 8= Back pain

65. For how long did you take the treatment? |__| TRTD
1= one month, 2= 2-6 months, 3= 7-12 months, 4= more than 12 months

66. Are you still taking the treatment? |__| TRTC
1 = yes, 2 = no
67. Are you currently taking any pain killers?
1 = yes, 2 = no **If no go to question 69** |__| PRT1

If yes,

68. How long have you been taking the pain killers?
1= one month, 2= 2-6 months, 3= 7-12 months, 4= more than 12 months |__| PRT2

69. Please list all the current medicines participant is taking. (Tick all that apply)
a. ACE/ARB Enalpril, Captopril, Lisonopril or Lorsartan/ Ibersatan etc |__| CRT1
b. Beta blockers eg propranolol; atenolol, carvedilol, bisoprolol etc |__| CRT2
c. Calcium channel blocker eg Nifedipine/ Amilodipine/Adalat XL |__| CRT3
d. Diuretics; Bendrofluazide/ Hydrochlorothalidone Lasix/ other diuretic |__| CRT4
e. NSAIDS, asprin, diclofenac, ibubrofen, aceclofenac etc |__| CRT5
f. HAART; TDF/ AZT, 3TC. ABC. NVP. EFV, PIs, |__| CRT6
p. Others specify……………………………….. |__| CRT7

PHYSICAL MEASUREMENTS (if not done, enter code 888)

70. Consent obtained for physical measurements?
1 = yes, 2 = no |__| CONSPHYS

Blood pressure (mm Hg) and Pulse

71. Time blood pressure taken: (HH:MM) |__|__|:|__|__|BPT

72. Blood pressure measured on right arm |__|BPARM
1 = yes, 2 = no
If it is not possible to use the right arm and the left arm is used, state reason
|__________________________|REASNARM

73. Arm circumference (cm) |__|__| AC

If arm circumference is under 24 cm use paediatric cuff size; if 24 – 32 cm use regular arm cuff size; if 33 – 41 cm use large arm cuff size; or if over 41 cm use thigh cuff size

74. systolic/diastolic blood pressure (mm Hg) and pulse (number / minute) **-** *take 3 readings*

1st systolic |__|__|__| SYST1 1st diastolic |__|__|__|DIAST1 1st pulse |__|__|__|PLS1

2nd systolic |__|__|__| SYST2 2nd diastolic |__|__|__|DIAST2 2nd pulse |__|__|__|PLS2

3rd systolic |__|__|__| SYST3 3rd diastolic |__|__|__|DIAST3 3rd pulse |__|__|__|PLS3

Average of 2nd & 3rd|__|__|__| SYSTAVG |__|__|__|DIASTAVG |__|__|__| PLSAVG

Blood pressure comment |______________________________________| BPCOM

Anthropometry

75. Height (cm) |__|__|__|•|__| HT

State if hairdo prevents sliding part of measuring rod from pressing flat against head:

Height comment |______________________________________| HTCOM

76. Weight (kg) |__|__|__|•|__| WT

77. Waist circumference (cm) |__|__|__|•|__| WC1

|__|__|__|•|__| WC2

If there is a difference greater than 3cm between WC1 and WC2, measure a third time: |__|__|__|•|__| WC3

78. Hips circumference (cm) |__|__|__|•|__| HC1

|__|__|__|•|__| HC2

If there is a difference greater than 3cm between HC1 and HC2, measure a third time: |__|__|__|•|__| HC3

BLOOD AND URINE SAMPLES

79. Consent obtained for taking blood for screening for HIV, Hepatitis B, Hepatitis C, diabetes, cholesterol, biochemistry, full blood count, creatinine and for gene sequencing as well as urinalysis?

1 = yes, 2 = no |__| CONSBLD

80. Interviewer code of the person taking the blood sample if different from the interviewer |__|__|DINTCODE

|__|__|__|__|__|__|__|__|LABNO

81. 8.5ml with plain serum
1 = specimen obtained, 2 = specimen to be obtained later, 7 = refused, 9 = failed |__| VAC

82. 6ml with EDTA
1 = specimen obtained, 2 = specimen to be obtained later, 7 = refused, 9 = failed |__| EDTA

83. Would you like to know the result of this HIV test? |__|KVCT
1 = yes, 2 = no, 8 = don't know/not sure

84. Would you like to know your results for possible diabetes, high cholesterol and kidney function? |__| DCLRES
1 = yes, 2 = no, 8 = don't know/not sure

85. Consent obtained for taking blood and urine for future use and storage of blood and urine samples? |__| CONBLDG
1 = yes, 2 = no

TREATMENT
Instruction to interviewer: please record here if any treatment provided to participant on the spot

Diagnosis:

Treatment:
