# Supplementary material for: Impaired renal function in a rural Ugandan population cohort
Source: Wellcome Open Res. 2019 May 20;3:149. Originally published 2018 Nov 19. [Version 3] doi: 10.12688/wellcomeopenres.14863.3 (PMC6560494; doi:10.12688/wellcomeopenres.14863.3)
Supplement: Peer review history

# Peer review: Reviewer response for version 3

**DOI:** 10.21956/wellcomeopenres.16684.r35571

**Published:** 2019-06-11

**Authors:** Kajiru G Kilonzo

**Affiliations:** 1 Internal Medicine Department, Kilimanjaro Christian Medical Center, Moshi, Tanzania

No further comments or suggestions. The authors have addressed my comments adequately.

I have read this submission. I believe that I have an appropriate level of expertise to confirm that it is of an acceptable scientific standard.

# Peer review: Reviewer response for version 2

**DOI:** 10.21956/wellcomeopenres.16352.r34526

**Published:** 2019-01-17

**Authors:** Kajiru G Kilonzo

**Affiliations:** 1 Internal Medicine Department, Kilimanjaro Christian Medical Center, Moshi, Tanzania

Thank you for the opportunity to review this article again.

**General Comments**

 A well written article and very relevant problem being questioned. The use of a General Population Cohort which is well documented is an opportunity to follow them up further. Use of the CKD epi formula is a good addition among published data in Africa. This work could be indexed with some additional work.

 I still think the article has important information to share to the scientific community even though the disease has not been defined properly. Efforts to define the link between impaired kidney function and CKD which is the assumed disease could be further made. Why is impaired kidney function a poor man’s estimate of chronic kidney disease in a community study? I note that this is somewhat discussed. More is needed to justify using impaired kidney function and CKD interchangeably.

 One way of looking at this is from the following argument derived from the authors’ data. From the multivariable analysis hypertension and anaemia are likely manifestations of chronic kidney disease, so it is not unreasonable to presume that impaired kidney function is an estimate of CKD.

 The study highlights the importance of determining the best way of estimating CKD from the community. This statement could be more emphasized. This could be coming out as the discussion point instead of a study limitation.

**Abstract**

 Very representative of the work. Include a recommendation from your conclusion if possible.

**Introduction**

 This part explains very clearly the importance of CKD, the unavailability of quality data and the variability of impaired renal function.

 -          Again, in the flow of thoughts, the transition between CKD and impaired renal function has not been linked.

 -          The last sentence of the second paragraph “Late diagnosis………” appears to be an original idea/conclusion. Rural populations have lower prevalence of CKD. (Stanifer et al., 2015 ^1^). This could be included in the discussion as well.

 Otherwise some justification for doing the study (as stated above) are clear.

**Methods**

 As the last statement of the introduction emphasizes on high quality sampling methods, It could be clearer how the authors sampled 5979 individuals from the 22,000 population. What was the sampling method? The population is within a prospective cohort study. Is it not true that the data collected was cross sectional?

**Discussion**

 The prevalence found of 1.6% is significantly low compared to the global prevalence of CKD. This needs to be discussed in light of the definitions used. Impaired renal function vs CKD. Remember while you hypothesize that the rural population have more CKD, this cohort has very low levels of decreased eGFR. The strength would be in discussing this.

 The second paragraph delineates why the results are not comparable, why not use studies which used the same measure of impaired renal function? Furthermore, the use of MDRD to estimate the eGFR can not explain such a huge difference in prevalence. Again, this needs to come out.

 In the risk factor paragraph, the authors mention that the traditional risks are not seen. Is there CKDu?

**Conclusions**

 It was not clear from the study why more data on risk factors is needed.

I have read this submission. I believe that I have an appropriate level of expertise to confirm that it is of an acceptable scientific standard, however I have significant reservations, as outlined above.

## References

[1] StaniferJWJingBTolanSHelmkeNMukerjeeRNaickerSPatelU: The epidemiology of chronic kidney disease in sub-Saharan Africa: a systematic review and meta-analysis. *Lancet Glob Health*.2014;2(3) : 10.1016/S2214-109X(14)70002-6 e174-81 10.1016/S2214-109X(14)70002-6 25102850

# Response

**Published:** 2019-05-09

**Authors:** Laurie Tomlinson

**Affiliations:** LSHTM, UK

Dear Professor Kilonzu,

Thank you very much for taking time to review our paper. We believe we have made the necessary changes to highlight the difference between impaired renal function and chronic kidney disease. We have added more details on the selection criteria of the study population and addressed all the comments that where raised by the reviewer making this manuscript much more improved from the second version.

Please find below a detailed response to each of the issues raised.

**General Comments**

**A well written article and very relevant problem being questioned. The use of a General Population Cohort which is well documented is an opportunity to follow them up further. Use of the CKD epi formula is a good addition among published data in Africa. This work could be indexed with some additional work.**

**I still think the article has important information to share to the scientific community even though the disease has not been defined properly. Efforts to define the link between impaired kidney function and CKD which is the assumed disease could be further made. Why is impaired kidney function a poor man’s estimate of chronic kidney disease in a community study? I note that this is somewhat discussed. More is needed to justify using impaired kidney function and CKD interchangeably.**

**One way of looking at this is from the following argument derived from the authors’ data. From the multivariable analysis hypertension and anaemia are likely manifestations of chronic kidney disease, so it is not unreasonable to presume that impaired kidney function is an estimate of CKD.**

**The study highlights the importance of determining the best way of estimating CKD from the community. This statement could be more emphasized. This could be coming out as the discussion point instead of a study limitation. Exactly as you have been saying**

Response

Thank you for the kind comments. You raise a very important issue on the distinction between impaired renal function and CKD. We have now further made this distinction in our paper. Please see page 7 under the section of definition of renal function.

We have also moved the issue of a single creatinine for community studies to the discussion section and made more suggestions for the same. Please see page 14 under the discussion section.

**Abstract**

**Very representative of the work. Include a recommendation from your conclusion if possible.**

Response

We have now advocated for more population based studies to characterize CKD. Please see recommendation under the conclusion section.

**Introduction**

**This part explains very clearly the importance of CKD, the unavailability of quality data and the variability of impaired renal function.**

**-          Again, in the flow of thoughts, the transition between CKD and impaired renal function has not been linked.**

**-          The last sentence of the second paragraph “Late diagnosis………” appears to be an original idea/conclusion. Rural populations have lower prevalence of CKD. (Stanifer et al., 2015** **^1^** **). This could be included in the discussion as well.**

**Otherwise some justification for doing the study (as stated above) are clear.**

Response

We have added a sentence to link impaired renal function and CKD. Please see page 4.

We have included the issue of rural vs urban prevalence of CKD in the discussion. Please see page 14 in the discussion section.

**Methods**

**As the last statement of the introduction emphasizes on high quality sampling methods, It could be clearer how the authors sampled 5979 individuals from the 22,000 population. What was the sampling method? The population is within a prospective cohort study. Is it not true that the data collected was cross sectional?**

Response

This has been further explained in the methods section.

*All eligible participants were evaluated for the study with an acceptance rate of 98%. A total of 6,397 participants were identified in the two rounds the GPC (2011–2012 and 2014–2015 with 5,979 (93.5%) individuals having valid creatinine results. The 418 who did not have valid creatinine did not differ significantly from those selected.*

The 22,000 is the general population with only 48% older than 13 years of age. Therefore, the eligible population for the study with 18 years and older was less than 8,000 people.  Furthermore, only participants available during the period of the survey are evaluated and included in studies.

**Discussion**

**The prevalence found of 1.6% is significantly low compared to the global prevalence of CKD. This needs to be discussed in light of the definitions used. Impaired renal function vs CKD. Remember while you hypothesize that the rural population have more CKD, this cohort has very low levels of decreased eGFR. The strength would be in discussing this.**

**The second paragraph delineates why the results are not comparable, why not use studies which used the same measure of impaired renal function? This is fair enough. Were there papers in Stanifer that used CKD epi on population samples? Furthermore, the use of MDRD to estimate the eGFR cannot explain such a huge difference in prevalence. Again, this needs to come out.**

**In the risk factor paragraph, the authors mention that the traditional risks are not seen. Is there CKDu?**

Response

Thank you for bringing these comments. We have modified the discussion to explain the lower than expected levels of impaired renal function. We have also cited studies that looked at impaired renal function which is actually most of the studies coming from SSA because they usually only do one measurement of creatinine and or microalbinuria. In the paper we say:

*“We found a lower prevalence of* *of eGFR <60 mL/min per 1.73 m ^2^ in this rural setting than would be expected according to previous studies ^3, 23^. This unexpected finding could be explained by the characteristics of the population under study. Over 66% of our study participants were less than 45 years of age yet CKD prevalence increases with age. The traditional risk factors for CKD like diabetes mellitus, hypertension, obesity, alcohol intake and smoking were quite low in our population (see table 1).”*

Regarding the possibility of CKDu, we cannot be certain. Certainly our estimated prevalence is far lower than that seen in hot spots like lowland regions in Sri Lanka, Nicaragua, and El Salvador. Our collaborators in Malawi have found similar urban and rural prevalence, making a disease related to agricultural work unlikely (paper under review). Although we do not know the cause of CKD in our population we cannot be confident that it represents CKDu. This is however a good consideration that requires further exploration.

**Conclusions**

**It was not clear from the study why more data on risk factors is needed. He is right to point out that this is always a bit of a cop out. Need a stronger finishing sentence.**

Response

This has been modified accordingly please see the conclusion section.

*We found that approximately one in five adults in rural Uganda had abnormal kidney function despite a low prevalence of diabetes and obesity. More population based studies are needed to further characterize kidney disease in SSA.*

# Peer review: Reviewer response for version 2

**DOI:** 10.21956/wellcomeopenres.16352.r34525

**Published:** 2019-01-04

**Authors:** John Feehally

**Affiliations:** 1 University of Leicester, Leicester, UK

The authors have responded very satisfactorily to the reservations I expressed.  The term ‘impaired renal function’ is now used rather than CKD since their methodology does not allow formal identification of CKD. The discussion is significantly improved in reviewing the limitations as well as strengths of the study.

 Two typos should be corrected:

 Discussion: Study Strengths & Limitations, para 2, line 6. The sentence should read: *“In addition our study was prospectively sampled from well people and is this **un**likely to be affected by any transient falls in eGFR associated with acute illness”*

 Discussion: Study Strengths & Limitations, para 2, line 8. The word *‘during’* should be deleted. The sentence should read: “….. *if blood tests are measured when people are unwell.”*

I have read this submission. I believe that I have an appropriate level of expertise to confirm that it is of an acceptable scientific standard.

# Peer review: Reviewer response for version 1

**DOI:** 10.21956/wellcomeopenres.16198.r34316

**Published:** 2018-12-17

**Authors:** Kajiru G Kilonzo

**Affiliations:** 1 Internal Medicine Department, Kilimanjaro Christian Medical Center, Moshi, Tanzania

This well written work has the aim to alert us of the presence of kidney diseases in a rural cohort. Even though the introduction could be built towards the question of the risk factors in view of CKD unknown, the idea is to provide us with a starting point for epidemiological discussion in rural Africa.

 The methodology has the strength of measuring serum creatinine using a standardized assay. And CKD Epi formula is an advantage. The main weakness is the use of one serum creatinine which actually brings up an ethical question in this General Population Cohort (1989, UK funded) especially for those found to have CKD. A report on what happened to them would be helpful. And hence the use of impaired renal function would fit more appropriately instead of CKD.

I have read this submission. I believe that I have an appropriate level of expertise to confirm that it is of an acceptable scientific standard, however I have significant reservations, as outlined above.

# Response

**Published:** 2018-12-18

**Authors:** Laurie Tomlinson

**Affiliations:** LSHTM, UK

Dear Professor Kilonzo,

Many thanks for your review of our paper. You raise very similar concerns to Professor Feehally and we agree with the issues you have raised. We hope that our new draft of the paper fully addresses these concerns. 

We very much agree about the importance of understanding renal and other outcomes among the cohort we have examined here. This work is underway as part of the ARK study (http://blogs.lshtm.ac.uk/ark/) and we will be able to report on changes in renal function and mortality rates at different levels of renal function when this work is complete.

# Peer review: Reviewer response for version 1

**DOI:** 10.21956/wellcomeopenres.16198.r34314

**Published:** 2018-12-03

**Authors:** John Feehally

**Affiliations:** 1 University of Leicester, Leicester, UK

This is in many ways a very well-executed population cohort study of adults in rural Uganda, with the aim of identifying chronic kidney disease (CKD) and associated risk factors. The organisational and logistic challenges of such a cohort study in rural sub-Saharan Africa are well understood, and the authors are to be congratulated for their achievement. The methods, result and discussion are all very clearly written.

 However the study has one fundamental design flaw.  CKD is a chronic condition, and by definition can only be confirmed when serum creatinine is measured, and from that GFR is estimated, on two separate occasions. Prevalence can be misleading when a single measurement is used.

 The authors themselves recognise this in the final sentence of ‘Study Strengths & Limitations’:   “We only measured creatinine on one occasion; two screenings are required for the formal definition of CKD.”

 Regrettably a substantial proportion of the CKD epidemiology literature is flawed by reliance on a single measurement of kidney function, and claiming to identify CKD. This is particularly so in studies from low and middle income countries.

 The challenge of recalling a study cohort for second sampling is recognised and understood, especially in a low resource setting, such as Uganda. But in the opinion of this reviewer does not justify the report here submitted. While the majority of those identified in this study are likely to have CKD, it is not sound to report these data as showing the prevalence of CKD, when a fundamental aspect of the epidemiological definition of CKD has not been accounted for.

I have read this submission. I believe that I have an appropriate level of expertise to state that I do not consider it to be of an acceptable scientific standard, for reasons outlined above.

# Response

**Published:** 2018-12-14

**Authors:** Laurie Tomlinson

**Affiliations:** LSHTM, UK

Dear Professor Feehally, 

Many thanks for your helpful comments on the first draft of this paper. While the vast majority of epidemiological surveys are based on one measure of creatinine, we fully agree that this is limited and indeed had tried to make this point. We have now fully revised the paper in line with your suggestions to make our approach and the limitations clear. We have also discussed in more depth the need for better quality research into the prevalence of kidney disease in sub-Saharan Africa in terms of population sampling, creatinine measurement and understanding how best to estimate GFR from creatinine levels in this population. This paper provides the first stage of a large ongoing study to do exactly this: http://blogs.lshtm.ac.uk/ark/ .We hope that this draft will now meet your approval.
